# Supplementary material for: Heat adaptation of phage T7 under an extended genetic code
Source: Virus Evol. 2021 Dec 1;7(2):veab100. doi: 10.1093/ve/veab100 (PMC8923235; doi:10.1093/ve/veab100)
Supplement: veab100_Supp [file veab100_supp.zip › Sequencing_Analysis.zip!/Sequencing_Analysis/A3/evidence/JC_66.html]

BRESEQ :: Evidence


| Predicted mutation | | | | | | |
| --- | --- | --- | --- | --- | --- | --- |
| evidence | seq id | position | mutation | annotation | gene | description |
| MC JC | NC\_001604.1 | 13,278 | Δ1,019 bp | intergenic (–/–) | – / – | –/– |

| Missing coverage evidence... | | | | | | | | | | |
| --- | --- | --- | --- | --- | --- | --- | --- | --- | --- | --- |
|  |  |  | seq id | start | end | size | ←reads | reads→ | gene | description |
| \* | \* | ÷ | NC\_001604.1 | 13278 | 14296 | 1019 | 1612 [523] | [14] 1100 | *–/–* | –/– |

| New junction evidence | | | | | | | | | | | |
| --- | --- | --- | --- | --- | --- | --- | --- | --- | --- | --- | --- |
|  | | seq id | position | reads (cov) | reads (cov) | score | skew | freq | annotation | gene | product |
| \* | ? | NC\_001604.1 | = 13277 | 522 (0.300) | 1073 (0.630) | 291/586 | 0.3 | 80.4% | intergenic (–/–) | *–/–* | –/– |
|  | ? | NC\_001604.1 | 14297 = | 14 (0.010) |  |  |  |  | intergenic (–/–) | *–/–* | –/– |

Only 100 of 1108 total aligned reads displayed.  

`AGACAAAGGTAAAGCACATGAGGAAGGTCGCCCCGTTTCTATTACTGACCTACGTGGTTCTGGCGCACTACGCCAACTATCTGATACTATTATTGCCCTTGAGCGTAATCAGCAAGGCGATATGCCTAACCTTGTCCTCGTTCGTATTCTCAAGTGCCGCTTTACTGGTGATACTGGTATCGCTGGCTACATGGAATACAACAAGGAAACCGGATGGCTTGAACCATCAAGTTACTCAGGGGAAGAAGAGTCACACTCAGAGTCAACAGACTGGTCCAACGACACTGACTTCTGACAGGATTCTTGA‑‑‑‑‑‑‑‑‑‑‑‑‑‑‑‑‑‑‑‑‑‑‑‑‑‑‑‑‑‑‑‑‑‑‑‑‑‑‑‑‑‑‑‑‑‑‑‑‑‑‑‑‑‑‑‑‑‑‑‑‑‑‑‑‑‑‑‑‑‑‑‑‑‑‑‑‑‑‑‑‑‑‑‑‑‑‑‑‑‑‑‑‑‑‑‑‑‑‑‑‑‑‑‑‑‑‑‑‑‑‑‑‑‑‑‑‑‑‑‑‑‑‑‑‑‑‑‑‑‑‑‑‑‑‑‑‑‑‑‑‑‑‑‑‑‑‑‑‑‑‑‑‑‑‑‑‑‑‑‑‑‑‑‑‑‑‑‑‑‑‑‑‑‑‑‑‑‑‑‑‑‑‑‑‑‑‑‑‑‑‑‑‑‑‑‑‑‑‑‑‑‑‑‑‑‑‑‑‑‑‑‑‑‑‑‑‑‑‑‑‑‑‑‑‑‑‑‑‑‑‑‑‑‑‑‑‑‑‑‑‑‑‑‑‑‑‑‑‑‑‑‑‑‑‑‑‑‑‑‑‑‑‑‑‑‑‑‑‑‑‑‑‑‑‑‑‑‑‑‑‑‑‑‑‑‑‑‑‑‑‑‑‑‑‑‑  >  NC_001604.1/12971‑13277`  
`‑‑‑‑‑‑‑‑‑‑‑‑‑‑‑‑‑‑‑‑‑‑‑‑‑‑‑‑‑‑‑‑‑‑‑‑‑‑‑‑‑‑‑‑‑‑‑‑‑‑‑‑‑‑‑‑‑‑‑‑‑‑‑‑‑‑‑‑‑‑‑‑‑‑‑‑‑‑‑‑‑‑‑‑‑‑‑‑‑‑‑‑‑‑‑‑‑‑‑‑‑‑‑‑‑‑‑‑‑‑‑‑‑‑‑‑‑‑‑‑‑‑‑‑‑‑‑‑‑‑‑‑‑‑‑‑‑‑‑‑‑‑‑‑‑‑‑‑‑‑‑‑‑‑‑‑‑‑‑‑‑‑‑‑‑‑‑‑‑‑‑‑‑‑‑‑‑‑‑‑‑‑‑‑‑‑‑‑‑‑‑‑‑‑‑‑‑‑‑‑‑‑‑‑‑‑‑‑‑‑‑‑‑‑‑‑‑‑‑‑‑‑‑‑‑‑‑‑‑‑‑‑‑‑‑‑‑‑‑‑‑‑‑‑‑‑‑‑‑‑‑‑‑‑‑‑‑‑‑‑‑‑‑‑‑‑‑‑‑‑‑‑‑‑‑‑‑‑‑‑‑‑‑‑‑‑‑‑‑‑‑‑‑‑‑‑‑‑‑‑ttcttgaCAGTTGTTTCATATGAAGAGATTGTTAAGTCACGATAATCAATAGGAGAAATCAATATGATCGTTTCTGACATCGAAGCTAACGCCCTCTTAGAGAGCGTCACTAAGTTCCACTGCGGGGTTATCTACGACTACTCCACCGCTGAGTACGTAAGCTACCGTCCGAGTGACTTCGGTGCGTATCTGGATGCGCTGGAAGCCGAGGTTGCACGAGGCGGTCTTATTGTGTTCCACAACGGTCACAAGTATGACGTTCCTGCATTGACCAAACTGGCAAAGTTGCAATTGAACCGAGAG  >  NC_001604.1/14297‑14592`  

                                                                                                                                                                                                                                                                                                                                                                                                                                                                                                                                                                                                                             
 `GACAAAGGTAAAGCACATGAGGAAGGTCGCCCCGTTTCTATTACTGACCTACGTGGTTCTGGCGCACTACGCCAACTATCTGATACTATTATTGCCCTTGAGCGTAATCAGCAAGGCGATATGCCTAACCTTGTCCTCGTTCGTATTCTCAAGTGCCGCTTTACTGGTGATACTGGTATCGCTGGCTACATGGAATACAACAAGGAAACCGGATGGCTTGAACCATCAAGTTACTCAGGGGAAGAAGAGTCACACTCAGAGTCAACAGACTGGTCCAACGACACTGACTTCTGACAGGATT                                                                                                                                                                                                                                                                                                               >  1:29417/1‑301`  
 `GACAAAGGTAAAGCACATGAGGAAGGTCGCCCCGTTTCTATTACTGACCTACGTGGTTCTGGCGCACTACGCCAACTATCTGATACTATTATTGCCCTTGAGCGTAATCAGCAAGGCGATATGCCTAACCTTGTCCTCGTTCGTATTCTCAAGTGCCGCTTTACTGGTGATACTGGTATCGCTGGCTACATGGAATACAACAAGGAAACCGGATGGCTTGAACCATCAAGTTACTCAGGGGAAGAAGAGTCACACTCAGAGTCAACAGACTGGTCCAACGAACCTGACTTCTGACAGGATT                                                                                                                                                                                                                                                                                                               >  1:85395/1‑301`  
       `GGTAAAGCACATGAGGAAGGTCGCCCCGTTTCTATTACTGACCTACGTGGTTCTGGCGCACTACGCCAACTATCTGATACTATTATTGCCCTTGAGCGTAATCAGCAAGGCGATATGCCTAACCTTGTCCTCGTTCGTATTCTAAAGTGCCGCTTTACTGGTGATACTGGTATCGCTGGCTACATGGAATACAACAAGGAAACCGGATGGCTTGAACCATCAAGTTACTCAGGGGAAGAAGAGTCACACTCAGAGTCAACAGACTGGTCCAACGACACTGACTTCTTACAGGATTCTTGAC                                                                                                                                                                                                                                                                                                         >  1:159070/1‑301`  
             `GCACATGAGGAAGGTCGCCCCGTTTCTATTACTGACCTACGTGGTTCTGGCGCACTACGCCAACTATCTGATACTATTATTGCCCTTGAGCGTAATAAGCAAGGCGATATGCCTAACCTTGTCCTCGTTCGTATTCTCAAGTGCCGCTTTACTGGTGATACTGGTATCGCTGGCTACATGGAATACAACAAGGAAACCGGATGGCTTGAACCATCAAGTTACTAAGGGGAAGAAGAGTCACACTCAGAGTCAACAGACTGTTCCAACGACACTGACTTCTGACAGGTTTCTTTACCGTTGT                                                                                                                                                                                                                                                                                                   >  1:19303/1‑301`  
               `ACATGAGGAAGGTCGCCCCGTTTCTATTACTGACCTACGTGGTTCTGGCGCACTACGCCAACTATCTGATACTATTATTGCCCTTGAGCGTAATCAGCAAGGCGATATGCCTAACCTTGTCCTCGTTCGTATTCTCAAGTGCCGCTTTACTGGTGATACTGGTATCGCTGGCTACATGGAATACAACAAGGAAACCGGATGGCTTGAACCATCAAGTTACTCAGGGGAAGAAGAGTCACACTCAGAGTCAACAGACTGGTCCAACGACACTGACTTCTGACAGGTTTCTTGACAGTTGTTT                                                                                                                                                                                                                                                                                                 <  1:89763/301‑1`  
                  `TGAGGAAGGTCGCCCCGTTTCTATTACTGACCTACGTGGTTCTGGCGCACTACGCCAACTATCTGATACTATTATTGCCCTTGAGCGTAATCAGCAAGGCGATATGCCTAACCTTGTCCTCGTTCGTATTCTCAAGTGCCGCTTTACTGGTGATACTGGTATCGCTGGCTACATGGAATACAACAAGGAAACCGGATGGCTTGAACCATCCAGTTCCTCAGGGGAAGAAGAGTCACACTCAGAGTCAACAGACTGGTCCAACGACACTGACTTCTGACAGGCTTCTTGACAGTTGTTTCAT                                                                                                                                                                                                                                                                                              >  1:97242/1‑301`  
                  `TGAGGAAGGTCGCCCCGTTTCTATTACTGACCTACGTGGTTCTGGCGCACTACGCCAACTATCTGATACTATTATTGCCCTTGAGCGTAATCAGCAAGGCGATATGCCTAACCTTGTCCTCGTTCGTATTCTCAAGTGCCGCTTTACTGGTGATACTGGTATCGCTGGCTACATGGAATACAACAAGGAAACCGGATGGCTTGAACCATCAAGTTACTCAGGGGAAGAAGAGTCACACTCAGAGTCAACAGACTGGTCCAACGACACTGACTTCTGACAGGATTCTTGACAGTTGTTTCAT                                                                                                                                                                                                                                                                                              >  1:94324/1‑301`  
                        `AGGTCGCCCCGTTTCTATTACTGACCTACGTGGTTCTGGCGCACTACGCCAACTAGCTGATACTATTAGTGCCCTTGGGCGTAATCAGCAAGGCGATATGCCTAACCTTGTCCTCGTTCGTATTCTCAAGTGCCGCTTTACTGGTGATACTGGTATCGCTGGCTACATGGAATACAACAAGGAAACCGGATGGCTTGAACCATCAAGTTACTCAGGGGAAGAAGAGTCACACTCAGAGTCAACAGACTGGTCCAACGACACTGACTTCTGACAGGATTCTTGACAGTTGTTTCATATGAAG                                                                                                                                                                                                                                                                                        <  1:174475/301‑1`  
                                    `aaagagacagGACCTACGTGGTTCTGGCGCACTACGCCAACTATCTGATACTATTATTGCCCTTGAGCGTAATCAGCAAGGCGATATGCCTAACCTTGTCCTCGTTCGTATTCTCAAGTGCCGCTTTACTGGTGATACTGGTATCGCTGGCTACATGGAATACAACAAGGAAACCGGATGGCTTGAACCATCAAGTTACTCAGGGGAAGAAGAGTCACACTCAGAGTCAACAGACTGGTCCAACGACACTGACTTCTGACAGGATTCTTGACAGTTGTTTCATATGAAGAGATTGTTAAGT                                                                                                                                                                                                                                                                            <  1:208632/291‑1`  
                                    `TTCTATTACTGACCTACGTGGTTCTGTCGCACTACGCCAACTATCTGATACTATTATTGCCCTTGAGCGTAATCAGCAAGGCGATATGCCTAACCTTGTCCTCGTTCGTATTCTCAAGTGCCGCTTTACTGGTGATACTGGTATCGCTGGCTACATGGAATACAACAAGGAAACCGGATGGCTTGAACCATCAAGTTACTCAGGGGAAGAAGAGTCACACTCAGAGTCAACAGACTGGTCCAACGACACTGACTTCTGACAGGATTCTTGACAGTTGTTTCATATGAAGAGATTGTTAAGT                                                                                                                                                                                                                                                                            >  1:244187/1‑301`  
                                    `TTCTATTACTGACCTACGTGGTTCTGGCGCACTACGCCAACTATCTGATACTATTATTGCCCTTGAGCGTAATCAGCAAGGCGATATGCCTAACCTTGTCCTCGTTCGTATTCTCAAGTGCCGCTTTACTGGTGATACTGGTATCGCTGGCTACATGGAATACAACAAGGAAACCGGATGGCTTGAACCATCAAGTTACTCAGGGGAAGAAGAGTCACACTCAGAGTCAACAGACTGGTCCAACGACACTGACTTCTGACAGGATTCTTGACAGTTGTTTCATATGAAGAGATTGTTAAGT                                                                                                                                                                                                                                                                            >  1:80364/1‑301`  
                                    `TTCTATTACTGACCTACGTGGTTCTGGCGCACTACGCCAACTATCTGATACTATTATTGCCCTTGAGCGTAATCAGCAAGGCGATATGCCTAACCTTGTCCTCGTTCGTATTCTCAAGTGCAGCTTTACTGGTGATACTGGTATCGCTGGCTACATGGAATACAACAAGGAAACCGGATGGCTTGAACCATCAAGTTACTCAGGGGAAGAAGAGTCACACTCAGAGTCAACAGACTGGTCCAACGACACTGACTTCTGACAGGATTCTTGACAGTTGTTTCATATGAAGAGATTGTTAAGT                                                                                                                                                                                                                                                                            >  1:122035/1‑301`  
                                     `TCTATTACTGACCTACGTGGTTCTGGCGCACTACGCCAACTATCTGATACTATTATTGCCCTTGAGCGTAATCAGCAAGGCGATATGCCTAACCTTGTCCTCGTTCGTATTCTCAAGTGCCGCTTTACTGGTGATACTGGTATCGCTGGCTACATGAAATACAACAAGGAAACCGGATGGCTTGAACCATAAAGTTACTCAGGGGAAGAAGAGTCACACTCAGAGTCAACAGACTGGTCCAACGACACTGACTTCTGACAGGATTCTTGACAGTTGTTTCATATGAAGAGATTGTAAAGTC                                                                                                                                                                                                                                                                           >  1:270864/1‑301`  
                                                      `gagacagGGCGCACTACGCCAACTATCTGATACTATTATTGCCCTTGAGCGTAATCAGCAAGGCGATATGCCTAACCTTGTCCTCGTTCGTATTCTCAAGTGCCGCTTTACTGGTGATACTGGTATCGCTGGCTACATGGAATACAACAAGGAAACCGGATGGCTTGAACCATCAAGTTACTCAGGGGAAGAAGAGTCACACTCAGAGTCAACAGACTGGTCCAACGACACTGACTTCTGACAGGATTCTTGACAGTTGTTTCATATGAAGAGATTGTTAAGTCACGATAATCAATAGGAG                                                                                                                                                                                                                                                          <  1:233889/294‑1`  
                                                      `gGGTTCTGGCGCACTACGCCAACGATCTGATACTATTATTGCCCTTGAGCGTAATCAGCAAGGCGATATGCCTAACCTTGTCCTAGTTCGTATTCTCAAGTGCCGCTTTACTGGTGATACTGGTATCGCTGGCTACATGGAATACAACAAGGAAACCGGATGGCTTGAACCATCAAGTTACTCAGGGGAAGAAGAGTCACACTCAGAGTCAACAGACTGGTACAACGACACTGACTTCTGACAGGATTCTTGACAGTTGTTTCATATGAAGAGATTGTTAAGTCACGATAATCAATAGGAG                                                                                                                                                                                                                                                          <  1:149487/300‑1`  
                                                           `gggtgatcggagatgtgtataagagacagATTATTGCCCTTGAGCGTAATCAGCAAGGCGATATGCCTAACCTTGTCCTCGTTCGTATTCTCAAGTGCCGCTTTACTGGTGATACTGGTATCGCTGGCTACATGGAATACAAAAAGGAAACCGGATGGCTTGAACCATCAAGTTACTCAGGGGAAGAAGAGTCACACTCAGAGTCAACAGACTGGTCCAACGACACTGACTTCTGACAGGTTTCTTGACAGTTGTTTCATATGAAGAGATTGTTAAGTCACGATAATCAATAGGAGAAATC                                                                                                                                                                                                                                                     <  1:281361/272‑1`  
                                                           `cttGCGCACTACGCCAACTATCTGATACTATTATTGCCCGTGAGCGTAATCAGCAAGGCGATATGCCTAACCTTGTCCTCGTTCGTATTCTCAAGTGCCGCTTTACTAGTGATACTGGTATCGCTGGCTACATGGAATACAACAAGGAAACCGGATGGCTTGAACCATCAAGTTACTCAGGGGAAGAAGAGTCACACTCAGAGTCAACAGACTGGTCCAACGACACTGACTTCTGACAGGATTCTTGACAGTTGTTTCATATGAAGAGATTGTTAAGTCACGATAATCAATAGGAGAAATC                                                                                                                                                                                                                                                     <  1:284982/298‑1`  
                                                                        `aatgtgtatagagacagTTATTGCCCTTGAGCGTAATCAGCAAGGCGATATGCCTAACCTTGTCCTCGTTCGTATTCTCAAGTGCCGCTTTACTGGTGATACTGGTATCGCTGGCTACATGGAATACAACAAGGAAACCGGATGGCTTGAACCATCAAGTTACTCAGGGGAAGAAGAGTCACACTCAGAGTCAACAGACTGGTCCAACGACACTGACTTCTGACAGGATTCTTGACAGTTGTTTCATATGAAGAGATTGTTAAGTCACGATAATCAATAGGAGAAATCAATATGATCGTTT                                                                                                                                                                                                                                        <  1:340760/284‑1`  
                                                                        `CCAACTATCTGATACTATAAGTGCAGTGGTGCGTAATCAGAAAGGAGATATGGCTAACCTTGGCCTCGTTCGTATTAACAAGTGCCGATGTACTGGAGATACTGGTGTCGCTGGCTACATGGAATACAACAAGGAAACCGGATTGCTTGAACCATCAAGTTAATCAGGGGAAGAAGAGTCACGCTCAGAGTCAACAGACTGGTGCAACGACACTGCCTTCTGACAGGATTCTTGACAGTTGGTTCATATGAAGAGATTGTTAAGTCACGATAATCAATAGGAGAAATCAATATGATCGTTT                                                                                                                                                                                                                                        <  1:241029/301‑1`  
                                                                         `CAACTATCTGATACTATTATTGCCCTTGAGCGTAATCAGCAAGGCGATATGCCTAACCTTGTCCTCGTTCGTATTCTCAAGTGCCGCTTTACTGGTGATACTGGTATCGCTGGCTACATGGAATACAACAAGGAAACCGGATGGCTTGAACCATCAAGTTACTCAGGGGAAGAAGAGTCACACTCAGAGTCAACAGACTGGTCCAACGACACTGACTT‑‑‑‑‑‑‑‑‑‑‑‑TTGACAGTTGTTTCATATGAAGAGATTGTTAAGTCACGATAATCAATAGGAGAAATCAATATctgtctcttatacacatctcc                                                                                                                                                                                                                           >  1:369/1‑280`  
                                                                              `ATCTGATACTATTATTGCCCTTGAGCGTAATCAGCAAGGCGATATGCCTAACCTTGTCCTCGTTCGTATTCTCAAGTGCCGCTTTACTGGTGATACTGGTATCGCTGGCTACATGGATTACAACAAGGAAACCGGATGGCTTGAACCATCAAGTTACTCAGGGGAAGAAGAGTCACACTCAGAGTCAACAGACTGGTCCAACGACACTGACTTCTGACAGGATTCTTGACAGTTGTTTCATATGAAGAGATTGTTAAGTCACGATAATCAATAGGAGAAATCctgtctcttatacacatct                                                                                                                                                                                                                                  >  1:52140/1‑282`  
                                                                              `ATCTGATACTATTATTGCCCTTGAGCGTAATCAGCAAGGCGATATGCCTAACCTTGTCCTCGTTCGTATTCTCAAGTGCCGCTTTACTGGTGATACTGGTATCGCTGGCTACATGGAATACAACAAGGAAACCGGATGGCTTGAACCATCAAGTTACTCAGGGGAAGAAGAGTCACACTCAGAGTCAACTGACTGGTCCAACGACACTGACTTCTGACAGGATTCTTGACAGTTGTTTCATATGAAGAGATTGTTAAGTCACGATAATCAATAGGAGAAATCAATATGATCGTTTCTGACA                                                                                                                                                                                                                                  >  1:152617/1‑301`  
                                                                              `ATCTGATACTATTATTGCCCTTGAGCGTAATCAGCAAGGCGATATGCCTAACCTTGTCCTCGTTCGTATTCTCAAGTGCCGCTTTACTGATGATACTGGTATCGCTGGCTACATGGAATACAACAAGGAAACCGGATGGATTGAACCATCAAGTTACTCAGGGGAAGAAGAGTCACACTCAGAGTCAACAGACTGGTCCAACGACACTGACTTCTGACAGGATTCTTGACAGTTGTTTCATATGAAGAGATTGTTAAGTCACGATAATCAATAGGAGAAATCAATATGATCGTTTCTGACA                                                                                                                                                                                                                                  >  1:68400/1‑301`  
                                                                                `CTGATACTCTTATTGCCCTTGAGCGTAATCAGCAAGGCGATATGCCTAACCTTGTCCTCGTTCGTATTCTCAAGTGCCGCTTTACTGGTGATACTGGTATCGCTGGCTACATGGAATACAACAAGGAAACCGGATGGCTTGAACCATCAAGTTACTCAGGGGAAGAAGAGTCACACTCAGAGTCAACAGACTGGTCCAACGACACTGACTTCTGACAGGATTCTTGACAGTTGTTTCATATGAAGAGATTGTTAAGTCACGATAATCAATAGGAGAAATCAATATGAACGTTTCTGACATC                                                                                                                                                                                                                                >  1:297194/1‑301`  
                                                                                      `CTATTATTGCCCTTGAGCGTAATCAGCAAGGCGATATGCCTAACCTTGTCCTCGTTCGTATTCTCAAGTGCCGCTTTACTGGTGATACTGGTATCGCTGGCTACATGGAATACAACAAGGAAACCTGATGGCTGGAACCATCAAGTTACTCAGGGGAAGAAGAGTCACACACAGAGTCAACAGACTGGTCCAACGACCCTGACTTCTGACAGGATTCTTGACAGTTGTTTCATATGAAGAGATTGTTAAGTCACGATAATCAATAGGAGAAATCAATATGACCGTTTCTGACATCACAGCT                                                                                                                                                                                                                          >  1:342425/1‑301`  
                                                                                      `CTATTATTGCCCTTGAGCGTAATCAGCAAGGCGATATGCCTAACCTTGTCCTCGTTCGTATTCTCAAGTGCCGCTTTACTGGTGATACTGGTATCGCTGGCTACATGGAATACAACAAGGAAACCGGATGGCTTGAACCATCAAGTTACTCAGGGGAAGAAGAGTCACACTCAGAGTCAACAGACTGGTCCAACGACACTGACTTCTGACAGGATTCTTGACAGTTGTTTCATATGAATAGATTGTTAAGTCACGATAATCAATAGGAAAAATCAATATGATCGTTTCTGACATCGAAGCT                                                                                                                                                                                                                          >  1:221125/1‑301`  
                                                                                      `CTATTATTGCCCTTGAGCGTAATCAGCAAGGCGATATGCCTAACCTTGTCCTCGTTCGTATTCTCAAGTGCCGCTTTACTGGTGATACTGGTATCGCTGGCTACATGGAATACAACAAGGAAACCGGATGGCTTGAACCATCAAGTTACTCAGGGGAAGAAGAGTCACACTCAGAGTCAACAGACTGGTCCAACGACACTGACTTCTGACAGGATTCTTGACAGTTGTTTCATATGAAGAGATTGTTAAGTCACGATAATCAATAGGAGAAATCAATATGATCGTTTCTGACATCGAAGCT                                                                                                                                                                                                                          >  1:177885/1‑301`  
                                                                                      `CTATTATTGCCCTTGAGCGTAATCAGCAAGGCGATATGCCTAACCTTGTCCTCGTTCGTATTCTCAAGTGCCGCTTTACTGGTGATACTGGTATCGCTGGCTACATGGAATACAACAAGGAAACCGGATGGCTTGAACCATCAAGTTACTCAGGGGAAGAAGAGTCACACTCAGAGTCAACAGACTGGTCCAACGACACTGACTTCTGACAGGATTCTTGACAGTTGTTTCATATGAAGAGATTGTTAAGTCACGATAATCAATAGGAGAAATCAATATGATCGTTTCTGACATCGAAGCT                                                                                                                                                                                                                          >  1:288176/1‑301`  
                                                                                        `ATTATTGCCCTTGAGCGTAATCAGCAAGGCGATATGCCTAACCTTGTCCTCGTTCGTATTCTCAAGTGCCGCTTTACTGTTGATACTGGTATCGCTGGCTACATGGAATACAACAAGGAAACCGGATGGCTTGAACCATCAAGCTACTCAGGGGAAGAAGAGTCACACCCAGAGTCACAAGACTGATCCAACGACACTGACTTCTGACAGGATTCTTGACAGTTGTTTCATATGAAGAGATTGTTAAGTCACGATAATCAATAGGAGAAATCAATATGATCtgtctcttatacacatctcc                                                                                                                                                                                                                        >  1:216343/1‑281`  
                                                                                        `ATTATTGCCCTTGAGCGTAATCAGCAAGGCGATATGCCTAACCTTGTCCTCGTTCGTATTCTCAAGTGCCGCTTTACTGGTGATACTGGTATCGCTGGCTACATGGAATACAACAAGGAAACCGGATGGCTTGAACCATCAAGTTACTCAGGGGAAGAAGAGTCACACTCAGAGTCAACAGACTGGTCCAACGACACTGACTTCTGACAGGATTCTTGACAGTTGTTTCATATGAAGAGATTGTTAAGTCACGATAATCAATAGGAGAAATCAATATGATCGTTTCTGACATCGAAGCTAA                                                                                                                                                                                                                        >  1:21017/1‑301`  
                                                                                        `ATTATTGCCCTTGAGCGTAATCAGCAAGGCGATATGCCTAACCTTGTCCTCGTTCGTATTCTCAAGTGCCGCTTTACTGGTGATACTGGTATCGCTGGCTACATGGAATACAACAAGGAAACCGGATGGCTTGAACCATCAAGTTACTCAGGGGAAGAAGAGTCACACTCAGAGTCAACAGACTGGTCCAACGACACTGACTTCTGACAGGATTCTTGACAGTCGTTTCATATGAAGAGATTGTTAAGTCACGATAATCAATAGGAGAAATCAATATGATCGTTTCTGACATCGAAGCTAA                                                                                                                                                                                                                        >  1:224216/1‑301`  
                                                                                        `ATTATTGCCCTTGAGCGTAATCAGCAAGGCGATATGCCTAACCTTGTCCTCATTCGTATTCTCAAGTGCCGCTTTACTGGTGATACTGGTATCGCTGGCTACATGGAATACAACAAGGAAACCGGATGGCTTGAACCATCAAGTTACTCAGGGGAAGAAGAGTCACACTCAGAGTCAACAGACTGGTCCAACGACACTGACTTCTGACAGGATTCTTGACAGTTGTTTCATATGAAGAGATTGTTAAGTCACGATAATCAATAGGAGAAATCAATATGATCGTTTCTGACATCGAAGCTAA                                                                                                                                                                                                                        >  1:314599/1‑301`  
                                                                                         `TTATTGCCCTTGAGCGTAATCAGCAAGGCGATATGCCTAACCTTGTCCTCGTTCGTATTCTCAAGTGCCGCTTTACTGGTGATACTGGTATCGCTGGCTACATGGAATACAACAAGGAAACCGGATGGCTTGAACCATCAAGTTACTCAGGGGAAGAAGAGTCACACTCAGAGTCAACAGACTGGTCCAACGACACTGACTTCTGACAGGATTCTTGACAGTTGTTTCATATGAAGAGATTGTTAAGTCACGATAATCAATAGGAGAAATCAATATGATCGTTTCTGACATCGAAGCTAAC                                                                                                                                                                                                                       >  1:13866/1‑301`  
                                                                                                   `aacagGTAATCAGCAAGGCGATATGCCTAACCTTGTCCTCGTTCGTATTCTCAAGTGCCGCTTTACTGGTGATACTGGTATCGCTGGCTACATGGAATACAACAAGGAAACCGGATGGCTTGAACCATCAAGTTACTCAGGGGAAGAAGAGTCACACTCAGAGTCAACAGACTGGTCCAACGACACTGACTTCTGACAGGATTCTTGACAGTTGTTTCATATGAAGAGATTGTTAAGTCACGATAATCAATAGGAGAAATCAATATGATCGTTTCTGACATCGAAGCTAACACCCTCTTAG                                                                                                                                                                                                             <  1:166992/296‑1`  
                                                                                                   `TGAGCGTAATCAGCAAGGCGATATGCCTAACCTTGTCCTCGTTCGTATTCTCAAGTGCCGCTTTACTGGTGATACTGGTATCGCTGGCTACATGGAATACAACAAGGAAACCGGATGGCTTGAACCATCAAGTTACTCAGGGGAAGAAGAGTCACACTCAGAGTCAACAGACTGGTCCAACGACACTGACTTCTGACAGGATTCTTGACAGTTGTTTCATATGAAGAGATTGTTAAGTCACGATAATCAATAGGAGAAATCAATATGATCGTTTCTGACATCGAAGCTAACGCCCTCTTAG                                                                                                                                                                                                             <  1:71831/301‑1`  
                                                                                                    `GAGCGTAATCAGCAAGGCGATATGCCTAACCTTGTCCTCGTTCGTATTCTCAAGTGCCGCTTTACTGGTGATACTGGTATCGCTGGCTACATGGAATACAACAAGGAAACCGGATGGCTTGATCCATCAAGTTACTCAGGGGAAGAAGAGTCACACTCAGAGTCAACAGACTGGTCCAACGACACTGACTTCTGACAGGATTCTTGACAGTTGTTTCATATGAAGAGATTGTTAAGTCACGATAATCAATAAGAGAAATCAATATGATCGTTTCTGACATCGAAGCTAACGCCCTCTTAGA                                                                                                                                                                                                            >  1:100119/1‑301`  
                                                                                                     `gGCGTAATCAGCAAGGCGATATGCCTAACATTGTCCTCGTTCGTATTCTCAAGTGCCGCTTTACTGGTGATACTGGTATCGCTGGCTACATGGAATACAACAAGGAAACCGGATGGCTTGAACCATCAAGTTACTCAGGGGAAGAAGAGTCACACTCAGAGTCAACAGACTGGTCCAACGACACTGACTTCTGACAGGTTTCTTGACAGTTGTTTCATATGAAGAGATTGTTAAGTCACGATAATCAATAGGAGAAATCAATATGATCGTTTCTGACATCGAAGCTAACGCCCTCTTAGAG                                                                                                                                                                                                           <  1:264448/300‑1`  
                                                                                                     `gGCGTAATCAGCAAGGAGATATGCCTAACCTTGTCCTTGTTCGTATTCTCAAGTGCCGCTTTACTGGTGCTACTGGTATCGCTGGCTACATGGAATACAACAAGGAAACCGGATGGCTTGAACCATCAAGTTACTCAGGGTAAGAAGAGTCACACTCAGAGTCAACAGACTGGTCCAACGACACTGACTTCTGACAGGATTCTTGACAGTTGTTTCATATGAAGAGATTGTTAAGTCACGATAATCAATAGGAGAAATCAATATGATCGTTTCTGATATCGAAGCTAACGCCCTCTTAGAG                                                                                                                                                                                                           <  1:46440/300‑1`  
                                                                                                     `cagGTAATCAGCAAGGCGATATGCCTAACCTTGTCCTCGTTCGTATTCTCAAGTGCCGCTTTACTGGTGATACTGGTATCGCTAGCTACATGGAATACAACAAGGAAACCGGATGGCTTGAACCATCAAGTTACTCAGGGGAAGAAGAGTCACACTCAGAGTCAACAGACTGGTCCAACGACACTGACTTCTGACAGGATTCTTGACAGTTGTTTCATATGAAGAGATTGTTAAGTCACGATAATCAATAGGAGAAATCAATATGATCGTTTCTGACATCGAAGCTAACGCCCTCTTAGAG                                                                                                                                                                                                           <  1:290086/298‑1`  
                                                                                                     `AGCGTAATCAGCAAGGCGATATGCCTAACCTTGTCCTCGTTCGTATTCTCAAGTGCCGCTTTACTGGTGATACTGGTATCGCTTGCTACATGGAATACAACAAGGAAACCGGATGGCTTTAACCATCAAGTTACTCAGGGGAAGAAGAGTCACACTCAGAGTCAACAGACTGGTCCAACGACACTGACTTCTGACAGGATTCTTGACAGTTGTTTCATATGAAGAGATTGTTAAGTCACGATAATCAATAGGAGAAATCAATATGATCGTTTCTGACATCGAAGCTAACGCCCTCTTAGAG                                                                                                                                                                                                           <  1:105511/301‑1`  
                                                                                                      `GCGTAATCAGCAAGGCGATATGCCTAACCTTGTCCTCGTTCGTATTCTCAAGTGCCGCTTTACTGGTGATACTGGTATCGCTGGCTACATGGAATACAACAAGGAAACCGGATGGCTTGAACCATCAAGTTACTCAGGGGAAGAAGAGTCACACTCAGAGTCAACAGACTGGTCCAACGACACTGACTTCTGACAGGATTCTTGACAGTTGTTTCATATGAAGAGATTGTTAAGTCACGATAATCAATAGGAGAAATCAATATGATCGTTTCTGACATCGAAGCTAACGCCCTCTTAGAGc                                                                                                                                                                                                          >  1:65076/1‑300`  
                                                                                                       `aGTAAGCAGCAAGGCGAGATGCCTAACCTTGTCCTCGTTCGTATTCTCAAGTGCCGCTTTACTGGTGATACTGGTATCGCTGGCTACATGGAATACAACAAGGAAACAGGATGGCTTGAACCATCAAGTTACTCAGGGGAAGAAGAGTCACACTCAGAGTCAACAGACTGGTCCAATGACACTGACTTCTGACAGGATTCTTGACAGTTGTTTCATATGAAGAGATTGTTAAGTCACGATAATCAATAGGAGAAATCAATATGATCGTTTCTGACATCGAAGCTAACGCCCTCTTAGAGAG                                                                                                                                                                                                         <  1:183778/300‑1`  
                                                                                                        `GTACTCAGCAAGGCGATATGCCTAACCTTGTCCTCGTTCGTATTCTCAAGTGCCGCTTTACTGGTGATACTGGTATCGCTGGCTACATGGAATACAACAAGGAAACCGGATGGCTTGAACCATCAAGTTACTCAGGGGAAGAAGAGTCACACTCAGAGTCAACAGACTGGTCCAACGACACTAACTTCTGACAGGATTCTTGACAGTTGTTTCATATGAAGAGATTGTTAAGTCACGATAATCAATAGGAGAAATCAATATGATCGTTTCTGACATCGAAGCTAACGCCCTCTTAGAGctt                                                                                                                                                                                                        >  1:197424/1‑298`  
                                                                                                             `CAGCAAGGCGATATGCCTAACCTTGTCCTCGTTCGTATTCTCAAGTGCCGCTTTACTGGTGATACTGGTATCGCTGGCTACATGGAATACAACAAGGAAACCGGATGGCTTGAACCATCAAGTTACTCAGGGGAAGAAGAGTCACACTCAGAGTCAACAGACTGGTCCAACGACACTGACTTCTGACAGGATTCTTGACAGTTGTTTCATATGAAGAGATTGTTAAGTCACGATAATCAATAGGAGAAATCAATATGATCGTTTCTGACATCGAAGCTAACGCCCTCTTAGAGAGCGTCAC                                                                                                                                                                                                   >  1:48521/1‑301`  
                                                                                                                     `gGATAAGCCTAACCTTGTCCACGTTCGTATTCTCAAGTGCCGATTTACTGGGGATACTGGTATCGCTGGCTACATGGAATACAACAAGGAAACCGGATGGCTTGAACCATCAAGTTACTCAGGGGAAGAAGAGTCACACTCAGAGTCAACAGACTGGTCCAACGACACTGACTTCTGACAGGATTCTTGACAGTTGTTTCATATGAAGAGATTGTTAAGTCACGATAATAAATAGGAGAAATCAATATGATCGTTTCTGACATCAAAGCTAACGCCCTCTTAGAGAGCGTCACTAAGTTCC                                                                                                                                                                                           <  1:10265/300‑1`  
                                                                                                                         `tTGCCTAACCTTGTCCTCGTTCGTATTCTCAAGTGCCGCTTTACTGGTGATACTTGTATCGATGGCTACATGGAATACAACAAGGAAACCGGAAGGCTTGAACCATCAAGTTACTCAGGGGAAGAAGAGTCACACTCAGAGTCAACAGACGGGTCCAACGAAACTGACTTCTGACAGGATTCTTAACAGTTGTTTCATATGAAGAGATTGTTAAGTCACGATAATCAATAGGAGAAATCAATATGATCGTTTCTGACATCGAAGCTAACGCCATATTAGAGAGCGTCACTAAGTTCCACTG                                                                                                                                                                                       <  1:18093/300‑1`  
                                                                                                                          `TGCCTAACCTTGTCCTCGTTCGTATTCTCAAGTGCCGCTTTACTGGTGATACTGGTATCGCTGGCTACATGGAATACAACAAGGAAACCGGATGGCTTGAACCATCAAGTTACTCAGGGGAAGAAGAGTCACACTCAGAGTCAACAGACTGGTCCAACGACACTGACTTATGACAGGATTCTTGACAGTTGTTTCATATGAAGAGATTGTTAAGTCACGATAATCAATAGGAGAAATCAATATGATCGTTTCTGACATCGAAGCTAACGCCCTCTTAGAGctgtctcttatacacatctcc                                                                                                                                                                                      >  1:275427/1‑280`  
                                                                                                                               `AACCTTGTCCTCGTTCGTATTCTCAAGTGCCGCTTTACTGGTGATACTGGTATCGCTGGCTACATGGAATACAACAAGGAAACCGGATGGCTTGAACCATCAAGTTACTCAGGGGAAGAAGAGTCACACTCAGAGTCAACAGACTGGTCCAACGACACTGACTTCTGACAGGATTCTTGACAGTTGTTTCATATGAAGAGATTGTTAAGTCACGATAATCAATAGGAGAAATCAATATGATCGTTTCTGACATCGAAGCTAACGCCCTCTTAGAGAGCGTCACTAAGTTCCACTGCGGGGT                                                                                                                                                                                 <  1:187919/301‑1`  
                                                                                                                                     `tTCCTCGTTCGTATTCTCAAGTGCCGCTTTACTGGTGATACTGGTATCGCTGGCTACATGGAATACAACAAGGAAACCGGATGGCTTGAACCATCAAGTTACTCAGGGGAAGAAGAGTCACACTCAGAGTCAACAGACTGGTCCAACGACACTGACTTCTGACAGGATTCTTGACAGTTGTTTCATATGAAGAGATTGTTAAGTCACGATAATCAATAGGAGAAATCAATATGATCGTTTCTGACATCGAAGCTAACGCCCTCTTAGAGAGCGTCACTAAGTTCCACTGCGGGGTTATCTA                                                                                                                                                                           <  1:161723/300‑1`  
                                                                                                                                         `TCGTTCGTATTCTCAAGTGCCGCTTTACTGGTGATACTGGTATCGCTGGCTACATGGAATACAACAAGGAAACCGGATGGCTTGAACCATCAAGTTACTCAGGGGAAGAAGAGTCACGCTCAGAGTTAACAGACTGGTCCAACGACACTGACTTCTGACAGGATTCTTGACAGTTGTTTCATATGAAGAGATTGTTAAGTCACGATAATCAATAGGAGAAATCAATATGATCGTTTCTGACATCGAAGCTAACGCCCTCTTAGAGAGCGTCACTAAGTTCAACTGCGGGGTTATCTACGAC                                                                                                                                                                       <  1:145745/301‑1`  
                                                                                                                                          `CGTTCGTATGCTCAAGTGCCGCTTTACTGGTGATACTGGTATCGCTAGCTACATGGAATACAACAAGGAAACCGGATGGCTTGAACCATCAAGTTACTCAGGTGAAGAAGAGTCACACTCAGAGTCAACAGACTGGTCCAACGACACTGACTTCTGACAGGATTCTTGACAGTTGTTTCATATGAAGAGATTGTTAAGTCACGATAATCAATAGGAGAAATCAATATGATCGTTTCTGACATCGAAGCTAACGCCCTCTTAGAGAGCGTCACTAAGTTCCACTGCGGGGTTATCTACGACT                                                                                                                                                                      <  1:205569/301‑1`  
                                                                                                                                              `agatgtgtataagggacagTTACTGGTGATACTGGTATCGCTGGCTACATGGAATACAACAAGGAAACCGGATGGCTTGAACCATCAAGTTACTCAGGGGAAGAAGAGTAACACTCAGAGTCAACAGACTGGTCCAACGACACTGACTTCTGACAGAATTCTTGACAGTTGTTTCATATGAAGAGATTGTTAAGTCACGATAATCAATAGGAGAAATCAATATGATCGTTTCTGACATCGAAGCTAACGCCCTCTTAGAGAGCGTCACTAAGTTCAACTGCGGGGTTATCTACGACTACTC                                                                                                                                                                  <  1:302140/282‑1`  
                                                                                                                                               `GTATGCTCAAGTGCCGCTTTACTGGAGATACTGGTATCGCTGGCTACATGGAATACAACAAGGAAACCGGATGGCTTGAACCATCAAGTTACTCATGGGAAGAAGAGTCACACTCAGAGTCAACAGACTGGTCCAACGACACTGACTTCTGACAGGATTCTTGACAGTTGTTTCATAAGAAGAGATTGTTAAGTCACGATAATCAATAGGAGAAATCAATATGATCGTTTCTGACATCGAAGCTAACGCCCTCTTAGAGAGCGTCACTAAGTTCCACTGCGGGGTTATCTACGACTACTCC                                                                                                                                                                 <  1:42102/301‑1`  
                                                                                                                                                 `agTCTCAAGTGCCGATTTACTGGTGATACTGGTATCGCTGGCTACATGGAATACAACAAGGAAACCGGATGGCTTGAACCATCAAGTTACTCAGGGGAAGAAGAGTCACACTCAGAGTCAACAGACTGGTCCAACGACACTGACTTCTGACAGGATTCTTGACAGTTGTTTCATATGAAGAGATTGTTAAGTCACGATAATCAATAGGAGAAATCAATATGATCGTTTCTGACATCGAAGCTAACGCCCTCTTAGAGAGCGTCACTAAGTTCCACTGCGGGGTTATCTACGACTACTCCAC                                                                                                                                                               <  1:203395/299‑1`  
                                                                                                                                                 `ATTCTCAAGTGCCGCTTTACTGGTGATACTGGTATCGCTGGCTACATGGAATACAACAAGGAAACCGGATGGCTTGAACCATCAAGTTACTCAGGGGAAGAAGAGTCACACTCAGAGTCAACAGACTGGTCCAACGACACTGACTTCTGACAGGATTCTTGACAGTTGTTTCATATGAAGAGATTGTTAAGTCACGATAATCAATAGGAGAAATCAATATGATCGTTTCTGACATCGAAGCCAATGCCCTCTTAGAGAGCTTCACTAAGTTACACTGCGGGGTTATCTACGCCTACTCCAC                                                                                                                                                               >  1:218591/1‑301`  
                                                                                                                                                 `ATTATCAAGTGCCGCTTTACTGGTGATACTGGTATCGCTGGCTACATGGAATACAACAAGGAAACCGGATGGCTTGAACCATCAAGTTACTCAGGGGAAGAAGAGTCACACTCAGAGTCAACAGACTGGTCCAACGACACTGACTTCTGACAGGATTCTTGACAGTTGTTTCATATGAAGAGATTGTTAAGTCACGATAATCAATAGGAGAAATCAATATGATCGTTTCTGACATCGAAGCTAACGCCCTCTTAGAGAGCGTCACTAAGTTCCACTGCGGGGTTATCTACGACTACTCCAC                                                                                                                                                               <  1:40130/301‑1`  
                                                                                                                                                    `gatgtgtataagagacagGGTGATACTGGTATCGCTGGCTACATGGAATACAACAAGGAAACCGGATGGCTTGAACCATCAAGTTACTCAGGGGAAGAAGAGTCACACTCAGAGTCAACAGACTGGTCCAAAGACACTGACTTCTGACAGGATTCTTGACAGTTGTTTCATATGAAGAGATTGTTAAGTCACGATAATCAATAGGAGAAATCAATATGATCGTTTCTGACATCGAAGCTAACGCCCTCTTAGAGAGCGTCACTAAGTTCCACTGCGGGGTTATCTACGACTACTACACCGC                                                                                                                                                            <  1:272760/283‑1`  
                                                                                                                                                    `CTCAAGTGCCGCTTTACTGGTGATACTGGTATCGCTGGCTACATGGAATACAACAAGGAAACCGGATGGCTTGAACCATCAAGTTACTCAGGGGAAGAAGAGTCACACTCAGAGTCAACAGACTGGTCCAACGACACTGACTTCTGACAGGATTCTTGACAGTTGTTTCATATGAAGAGATTGTTAAGTCACGATAATCAATAGGAGAAATCAATATGATCGTTTCTGACATCGAAGCTAACGCCCTCTTAGAAACCGTCACTAATTTCAACTGCTGGGTTATCACCGACTACCCCCCCcg                                                                                                                                                            >  1:775/1‑299`  
                                                                                                                                                    `CTCAAGTGCCGCTTTACTGGTGATACTGGTATCGCTGGCTACATGGAATACAACAAGGAAACCGGATGGCTTGAACCATCAAGTTACCCAGGGGAAGAAGAGTCACACTCAGAGTCAACAGACTGGTCCAACGACACTGACTTCTGACAGGATTCTTGACAGTTGTTTCATATGAAGAGATTGTTAAGTCACGATAATCAATAGGAGAAATCAATATGATCGTTTCTGACATCGAAGCTAACGCCCTCTTAGAGAGCGTCACTAAGTTCCACTGCGGGGTTATCTACGACTACTCCAACGC                                                                                                                                                            >  1:73230/1‑301`  
                                                                                                                                                                `TTTACTGGTGATACTGGTATCGCTAGCTACATGGAATACAACAAGGAAACCGGATGGCTTGAACCATCAAGTTACTCAGGGGAAGAAGAGTCACACTCAGAGTCAACATACTGGTCCAACGACACTGACTTCTGACAGGATTCTTGACAGTTGTTTCATATGAAGAGATTGTTAAGTCACGATAATCAATAGGAGAAATCAATATGATCGTTTCTGACATCGAAGCTAACGCCCTCTTAGAGAGCGTCACTAAGTTCCACTGCGGGGTTATCTACGACTACTCCACCGCTGAGTACGTAAG                                                                                                                                                <  1:331796/301‑1`  
                                                                                                                                                                    `CTGGTGATACTGGTATCGCTGGCTACATGGAATACAACAAGGAAACCGGATGGCTTGAACCATCAAGTTACTCAGGGGAAGAAGAGTCACACTCAGAGTCAACAGACTGGTCCAACGACACTGACTTCTGACAGGATTCTTGACAGTTGTTTCATATGAAGAGATTGTTAAGTCACGATAATCAATAGGAGAAATCAATATGATCGTTTCTGACATAGAAGCTAACGCCCTCTTAGAGAGCGTCACTAAGTTCCACTGCGGGGTTATCTACGACTACTCCACCGCTGAGTACGTAAGCTAC                                                                                                                                            >  1:132257/1‑301`  
                                                                                                                                                                      `GGTGATACTGGTATCGCTGGCTACATGGAATACAACAAGGAAACCGGATGGCTTGAACCATCAAGTTACTCAGGGGAAGAAGAGTCACACTCAGAGTCAACAGACTGGTCCAACGACACTGACTTC‑‑‑‑‑‑‑‑‑‑‑‑TGACAGTTGTTTCATATGAAGAGATTGTTAAGTCACGATAATCAATAGGAGAAATCAATATGATCGTTTCTGACATCGAAGCTAACGCCCTCTTAGAGAGCGTCACTAAGTTCCACTGCGGGATTATCTACGACTACTCCACCGCTGAGTCCGTAAGCTACCGTCCGAGTGACTT                                                                                                                              >  1:250409/1‑301`  
                                                                                                                                                                       `GTGATACTGGTATCGCTGGCTACATGGAATACAACAAGGAAACCGGATGGCTTGAACCATCAAGTTACTCATGGGAAGAAGAGTCACACTCAGAGTCAACAGACTGGTCCAACGACACTGACTTCTGACAGGTTTCTTGACAGTTGTTTCATATGAAGAGATTGTTAAGTCACGATAATCAATAGGAGAAATCAATATGATCGTTTCTGACATCGAAGCTAACGCCCTCTTAGAGAGCGTCACTAAGTTCCACTGCGGGGTTATCTACGACTACTCCACCGCTGAGTACGTAAGCTACCGT                                                                                                                                         >  1:3080/1‑301`  
                                                                                                                                                                          `ATACTGGTATCGCTGGCTACATGGAATACAACAAGGAAACCGGATGGCTTGAACCACCAAGCTACTCAGGGGAAGAAGAGTCACACTCAGAGTCAACAGACTGGTCCAACGACAACGACTTCTGACAGGATTCTTGACAGTTGTTTCATATGAAGAGATTGTTAAGTCACGATAACCAACAGGAGAAATCAATATGATCGTTTCTGACATCGAAGCTAACGCCCTCTTAGAGAGCGTCACTAAGTTCCACTGCGGGGTTATCTACGACTACTCCACCGCTGAGTACGTAAGCCACCGTACG                                                                                                                                      >  1:172320/1‑301`  
                                                                                                                                                                               `GGTATCGCTGGCTACATGGAATACAACAAGGAAACCGGATGGCTTGAACCATCAAGTTACTCAGGGGAAGAAGAGTCACACTCAGAGTCAACAGACTGGTCCAACGACACTGACTTCTGACAGGATTCTTGACAGTTGTTTCATATGAAGAGATTGTTAAGTCACGATAATCAATAGGAGAAATCAATATGATCGTTTCTGACATCGAAGCTAACGCCCTCTTAGAGAGCGTCACTAAGTTCCACTGCGGGGTTATCTACGACTACTCCACCGCTGAGTACGTAAGCTACCGTCCGAGTGA                                                                                                                                 >  1:157160/1‑301`  
                                                                                                                                                                               `GGTATCGCTGGCTACATGGAATACAACAAGGAAACCGGATGGCTTGAACCATCAAGTTACTCAGGGGAAGAAGAGTCACACTCAGAGTCAACAGACTGGTCCAACGACACTGACTTCTGACAGGATTCTTGACAGTTGTTTCATATGAAGAGATTGTTAAGTCACGATAATCAATAGGAGAAATCAATATGATCGTTTCTGACATCGAAGCTAACGCCCTCTTAGAGAGCGTCACTAAGTTCCACTGCGGGGTTATCTACGACTACTCCACCGCTGAGTACGTAAGCTACCGTCCGAGTGA                                                                                                                                 >  1:267655/1‑301`  
                                                                                                                                                                                `gctcggagatgtgtataagagACAGCAAGGAAACCGGATGGCTTGAACCATCAAGTTACTCATGGGAAGAAGAGTCACACTCAGAGTCAACAGACTGGTCCAACGACACTGACTTCTGACAGGATTCTTGACAGTTGTTTCATATGAAGAGATTGTTAAGTCACGATAATCAATAGGAGAAATCAATATGATCGTTTCTGACATCGAAGCTAACGCCCTCTTAGAGAGCGTCACTAAGTTCCACTGCGGGGTTATCTACGACTACTCCACCGCTGAGTACGTAAGCTACCGTCCGAGTGAC                                                                                                                                <  1:229440/280‑1`  
                                                                                                                                                                                       `gGGCTACATGGAATACAACAAGGAAACCGGATGGCTTGAACCATCAAGTTACTCAGGGGAAGAAGAGTCACACTCAGAGTCAACAGACTGGTCCAACGACACTGACTTCTGACAGGATTCTTGACAGTTGTTTCATATGAAGAGATTGTTAAGTCACGATAATCAATAGGAGAAATCAATATGATCGTTTCTGACATCGAAGCTAACGCCCTCTTAGAGAGCGTCACTAAGTTCCACTGCGGGGTTATCTACGACTACTCCACCGCTGAGTACGTAAGCTACCGTCCGAGTGACTTCAGTG                                                                                                                         <  1:14788/300‑1`  
                                                                                                                                                                                            `ACATGGAATACAACAAGGAAACCGGATGGCTTGAACCATCAAGTTACTCAGGGGAAGAAGAGTCACACTCAGAGTCAACAGACTGGTCCAACGACACTGACTTCTGACAGGATTCTTGACAGTTGTTTCATATGAAGAGATTGTTAAGTCACGATAATCAATAGGAGAAATCAATATGATCGTTTCTGACATCGAAGCTAACGCCCTCTTAGAGAGCGTCACTAAGTTCCACTGCGGGGTTATCTACGACTACTCCACCGCTGAGTACGTAAGCTACCGTCCtgtctcttatacacatctc                                                                                                                    >  1:169689/1‑282`  
                                                                                                                                                                                             `gagatgtgtataaggtacagCCGGATGTCTTGAACCATCAAGTTACTCAGGGGAAGAAGAGTCACACTCAAAGTCAACACACTGGTCCAACGACACTGACTTCTGACAGGATTCTTGACAGTTGTTTCATACGAAGAGATTGTTAAGTCACGATAATCAATAGGAGAAATCAATATGATCGTTTCTGACATCGAAGCTAACGCCCTCTTAGAGAGCGTCACTAAGTTCCACTGCGGGGTTATCTACGACTACTCCACCGCTGAGTACGTAAGCTACCGTCCGAGTGACTTCGGTGCGTATC                                                                                                                   <  1:118158/281‑1`  
                                                                                                                                                                                                 `GAATACAACAAGGAAACCGGATGGCTTGAACCATCAAGTTACTCAGGGGAATAAGAGTCACACTCAGAGTCAACAGACTGGTCCAACGACACTGACTTCTGACAGGATTCTTGACAGTTGTTTCATATGAAGAGATTGTTAAGTCACGATAATCAATAGGAGAAATCAATATGATCGTTTCTGACATCGAAGCTAACGCCCTCTTAGAGAGCGTCACTAAGTTCCACTGCGGTGTTATCTACGACTACTCCACCGCTGAGTACGTAAGCTACCGTCCGAGTGACTTCGGTGCGTATCTGGA                                                                                                               >  1:238182/1‑301`  
                                                                                                                                                                                                 `GAATACAACAAGGAAACCGGATGGCTTGAACCATCAAGTTACTCAGGGGAAGAAGAGTCACACTCAGAGTCAACAGACTGGTCCAACGACACTGACTTCTGACAGGATTCTTGACAGTTGTTTCATATGAAGAGATTGTTAAGTCACGATAATCAATAGGAGAAATCAATATGATCGTTTCTGACATCGAAGCTAACGCCCTCTTAGAGAGCGTCACTAAGTTCCACTGCGGGGTTATCTACGACTACTCCACCGCTGATTACGTAAGCTACCGTCCGAGTGACTTCGGTGCGTATCctgt                                                                                                               >  1:327467/1‑297`  
                                                                                                                                                                                                     `ACAACAAGGAAACCGGATGGCTTGAACCATCAAGTTACTCAGGGGAAGAAGAGTCACACTCAGAGTCAATAGACTGGTCCAACGACACTGACGTCTGACAGGATTCTTGACAGTTGTTTCATATGAAGAGATTGTTAAGTCACGATCATCTATAGGAGAAATCAATATGATCGTTTCTGACATCGAAGCTAACGCCCTCTTAGAGATCGTCACTACGTTCCACTGCGGGGTTCTCTACGACTACTCCACCGCTGAGTACGTACGCTACCGTCCGAGTGCCTTAGGTGCGTATCTGGCTGCG                                                                                                           >  1:22694/1‑301`  
                                                                                                                                                                                                                       `GGCTTGAACCATCAAGTTACTCATGGGAAGAAGAGACACACTCAGAGTCAACAGACTGGTCCAACGACACTGACTTCAGACAGGAATCTTGACAGTTGTTTCATATGAAGAGATTGTTAAGTCACGATAATCAATAGGAGAAAACAATATGATCGTTCCTGACATCGAAGCTACCGCCCTCTTAGAGAGCGTCACTAAGTTCCACTGCTGGGTTAACTACGACTACTACACCGCTGAGTACGTAAGCTACCGTCCGAGTGACTTCGGTGCGTAACTGGATGCGCTGGAAGACGAGGCTGtg                                                                                         >  1:155015/1‑299`  
                                                                                                                                                                                                                                    `AAGTTACTCAGGGGAAGAAGAGTCACACTCAGAGTCAACAGACTGGTCCAACGACACTGACTTCTGACAGGATTCTTGACAGTTGTTTCATATGAAGAGATTGTTAAGTCACGATAATCAATAGGAGAAATCAATATGATCGTTTCTGACATCGAAGCTAACGCCCTCTTAGAGAGCGTCACTAAGTTCCACTGCGGGGTTATCTACGACTACTCCACCGCTGAGTACGTAAGCTACCGTCCGAGTGACTTCGGTGCGTATCTGGATGCGCTGGAAGCCGAGGTTGCACGAGGCGGTCTTt                                                                            >  1:164206/1‑300`  
                                                                                                                                                                                                                                              `GGGGAAGAAGAGTCACACTCAGAGTCAACAGACTGGTCCAACGACACTGACTTCTGACAGGATTCTTGACAGTTGTTTCATATGAAGAGATTGTTAAGTCACGATAATCAATAGGAGAAATCAATATGATCGTTTCTGACATCGAAGCTAACGCCCTCTTAGAGAGCGTCACTAAGTTCCACTGCGGGGTTATCTACGACTACTCCACCGCTGAGTACGTAAGCTACCGTCCGAGTGACTTCGGTGCGTATCTGGATGCGCTGGAAGCCGAGGTTGCACGAGGCGGTCTTATTGTGTTCCA                                                                  >  1:259841/1‑301`  
                                                                                                                                                                                                                                              `GGGGAAGAAGAGTCACACTCAGAGTCAACAGACTGGTCCAACGACACTGACTTCTGACAGGATTCTTGACAGTTGTTTCATATGAAGAGATTGTTAAGTCACGATAATCAATAGGAGAAATCAATATGATCGTTTCTGACATCGAAGCTAACGCCCTCTTAGAGAGCGTCACTAAGTTCCACTGCGGGGTTATCTACGACTACTCCACCGCTGAGTACGTAAGCTACCGTCCGAGTGACTTCGGTGCGTATCTGGATGCGCTGGAAGCCGAGGTTGCACGAGGCGGTCTTATTGTGTTCCA                                                                  >  1:20078/1‑301`  
                                                                                                                                                                                                                                              `GGGGAAGAAGAGTCACACTCAGAGTCAACAGACTGGTCCAACGACACTGACTTCTGACAGGATTCTTGACAGTTGTTTCATATGAAGAGATTGTTAAGTCACGATAATCAATAGGAGAAATAAATATGATCGTTTCTGACATCGAAGCTAACGCCCTCTTAGAGAGCGTCACTAAGTTCCACTGCGGGGTTATCTACGACTACTCCACCGCTGAGTACGTAAGCTACCGTCCGAGTGACTTCGGTGCGTATCTGGATGCGCTGGAAGCCGAGGTTGCACGAGGCGGTCTTATTGTGTTCCA                                                                  >  1:135709/1‑301`  
                                                                                                                                                                                                                                               `tgtataagagacagCACTCAGAGTCAACAGACTGGTCCAACGACACTGACTTCTGACAGGATTCTTGACAGTTGTTTCATATGAAGAGATTGTTAAGTCACGATAATCAATAGGAGAAATCAATATGATCGTTTCTGACATCGAAGCTAACGCCCTCTTAGAGAGCGTCACTAAGTTCCACTGCGGGGTTATCTACGACTACTCCACCGCTGAGTACGTAAGCTACCGTCCGAGTGACTTCGGTGCGTATCTGGATGCGCTGGAAGCCGAGGTTGCACGAGGCGGTCTTATTGTGTTCCAC                                                                 <  1:212688/287‑1`  
                                                                                                                                                                                                                                               `GGGAAGAAGAGTCACACTCAGAGTCAACAGACTGGTCCAACGACACTGACTTCTGACAGGATTCTTGACAGTTGTTTCATATGAAGAGATTGTTAAGTCACGATAATCAATAGGAGAAATCAATATGATCGTTTCTGACATCGAAGCTAACGCCCTCTTAGAGAGCGTCACTAAGTTACACTGCGGGGTTATATACGACTACTCCACCGCTGAGTACGTAAGCTACCGTCCGAGTGACTTCGGTGCGTATCTGGATGCGCTGGAAGCCGAGGTTGCACGAGGCGGTCTTATTGTGTTCCAC                                                                 <  1:322830/301‑1`  
                                                                                                                                                                                                                                               `GGGAAGAAGAGTCACACTCAGAGTCAACAGACTGGTCCAACGACACTGACTTCTGACAGGATTCTTGACAGTTGATTCATATGAAGAGATTGTTAAGTCACGATAATCAATAGGAGAAATCAATATGATCGTTTCTGACATCGAAGCTAACGCCCTATTAGAGAGCGTCACTAAGTTCCACTGCGGGGTTATCTACGACTACTCCACCGCTGAGTACGTAAGCTACCGTCCGAGTGACTTCGGTGCGTATCTGGATGCGCTGGAAGCCGAGGTTGCACGAGGCGGTCTTATTGTGTTCCAC                                                                 <  1:190968/301‑1`  
                                                                                                                                                                                                                                                 `GAAGAAGAGTCACACTCAGAGTCAACAGACTGGTCCAACGACACTGACTTCTGACAGGATTCTTGACAGTTGTTTCATATGAAGAGATTGTTAAGTCACGATAATCAATAGGAGAAATCAATATGATCGTTTCTGACATCGAAGCTAACGCCCTCTTAGAGAGCGTCACTAAGTTCCACTGCGGGGTTATCTACGACTACTCCACCGCTGAGTACGTAAGCTACCGTCCGAGTGACTTCGGTGCGTATCTGGATGTGCTGGAAGCCGAGGTTGCACGAGGCGGTCTTATTGTGTTCCACAA                                                               >  1:115015/1‑301`  
                                                                                                                                                                                                                                                         `GTCACACTCAGAGTCAACAGACTGGTCCAACGACACTGACTTCTGACAGGATTCTTGACAGTTGTTTCATATGAAGAGATTGTTAAGTCACGATAATCAATAGGAGAAATCAATATGATCGTTTCTGACATCGAAGCTAACGCCCTCTTAGAGAGCGTCACTAAGTTCCACTGCGGGGTTATCTACGACTACTCAACCGCTGAGGACGTAAGATACCGTCCGAGTGACTTCGGTGCGTATCTGGATGCGCTGGAAGTCGAGGTTGCACGAGGCGGTGTTATTGTGTTCCACAACGGTCACA                                                       >  1:141054/1‑301`  
                                                                                                                                                                                                                                                           `aACACACAGAGTACACAGACTGGTCCAACGACACTGACTTCTGACAGGATTCTTGACAGTTGTTTCATCTGAAGAGATTGTTAAGTCACGATAATCAATAGGAGAAATCAATATGATCGTTTCTGAAATAGAAGCTACCGCCCTCTTAGAGAGCGTCACTATGTTCCACAGCGGGGTTATCTACGACTACTCCACCGCTGAGTACGTAAGCTACCGTCCGAGTGACTTCGGTACGTATCTGGCTGCGCTGGAAGCCGAGGTTGCACGAGGCGGTCTTATTGTGTTCCACAACGGTCACAAG                                                     <  1:257799/300‑1`  
                                                                                                                                                                                                                                                           `CACACTCAGAGTCAACAGACTGCTCCAACGACACTGACTTATGTCAGGATTCTTGACAGTTGTTTCATATGAAGAGATTGTTAAGTCACGATAATCAATAGGAGAAATCAATATGATCGTTTCTGACATCGAAGCCAACGCCATCTTAGAGAGCGTCACTAAGTGCCACTGCGGGGTTATCTACGACTACTCCACCGCTGAGTACGTAAGCTACCGTCCGAGTGACTTCGGTGCGTATCTGGATGCGCTGGAAGCCGAGGTTGCACGAGGCGGTCTTATTGTGTTCCACAACGGTCACAAG                                                     <  1:126627/301‑1`  
                                                                                                                                                                                                                                                             `CACTCAGAGTCAACAGACTGGTCCAACGACACTGACTTCTGACAGGATTCTTGACAGTTGTTTCATATGAAGAGATTGTTAAGTCACGATAATCAATATGAGAAATCAATATGATCGTTTCTGACATCGAAGCTAACGCCCTCTTAGAGAGCGTCACTAAGTTCCACTGCGGGGTTATCTACGACTACTCCACCGCTGAGTACGTAAGCTACCGTCCGAGTGACTTCGGTGCGTATCTGGATGCGCTGGAAGCCGAGGTTGCACGAGGCGGTCTTATTGTGTTCCtgtctcttatacacat                                                   >  1:338276/1‑285`  
                                                                                                                                                                                                                                                             `CACTCAGAGTCAACAGACTGGTCCAACGACACTGACTTCTGACAGGATTCTTGACAGTTGTTTCATATGAAGAGATTGTTAAGTCACGATAATCAATAGGAGAAATCAATATGAGCGTTTCTGACATCGAAGCTAACGCCCGCTTAGAGAGCGTCACTAAGTTCCACTGCGGGGTTATCTACTACTACTCCACCGCTGAGTACGTAAGCTACCGTCCGAGTGACTTCGGTGCGTATCTGGATGCGCTGGAAGCCGAGGTTGCACGAGGCGGTCTTATTGTGTTCCACAACGGTCACAAGTA                                                   <  1:57784/301‑1`  
                                                                                                                                                                                                                                                                `cagaagtagaatGACTGGTCCAACGAAACTAAGTTATAGCAGGATTCTTGAAAGTTGTTTCATATGAAGATATTGTTAAGTCACGATAAACAATAGGAGAAATCAAGATGATCGTTTCTGACATGGAAGCTAACGCCCTCTTAGAGAGCGTCACTAAGTTCCACTGCGGGGTTATCTACGACTACGCCACCGCTGAGTACGTAAGCTACCGTCCGAGTGACTTCGGTGCGTATCTAGATGCGCTGGAAGCCGAGGTTGCACGAGGCGTTCTTATTGTGTTCCACAACGGTCACAAGTATGA                                                <  1:311061/289‑1`  
                                                                                                                                                                                                                                                                `TCAGAGTCAACAGACTGGTCCAACGACACTGACTTCTGACAGGATTCTTGACAGTTGTTTCATATGAAGAGATTGTTAAGTCACGATAATCAATAGGAGAAATCAATATGATCGTTTCTGACATCGAAGCTAACGCCCTCTTAGAGAGCGTCACTAAGTTCCACTGCGGGGTTATCTACGACTACTCCACCGCTGAGTACGTAAGCTACCGTCCGAGTGACTTCGGTGCGTATCTGGATGCGCTGGAAGCCGAGGTTGCACGAGGCGGTCTTATTGTGTTCCACAACGGTCACAAGTATGA                                                <  1:87216/301‑1`  
                                                                                                                                                                                                                                                                 `CAGAGTCAACATACTGGTCCAACGACACTTACTTCTGACAGGATTCTTGACAGTTGTTTCATATGAAGATATTGTTAAGTCACGATAATCAATAGGAGAAATCAATATGATCGTTTCTGACATCGAAGCTAATGCCCTCTTAGAGAGCGTCACTAAGTTCCACTGCTGGGTTCTCTACGACTACTCCACCGCTGAGTACGTAAGCTACCGTCCGAGTGACTTCGGTGCGTATCTGGATGCGCTGGAAGCCGAGGTTGCACGAGGCGGTCTTATTGTGTTCCACAACGGTCACAAGTATGAC                                               <  1:50235/301‑1`  
                                                                                                                                                                                                                                                                   `GAGGCAACAGACTGGTCCAACGACACTGACTTCTGACAGGATTCTTGACAGTTGTTTCATATGAAGAGATTGTTAAGTCACGATAATCAATAGGAGAAATCAATATGATCGTTTCTGACATCGAAGCTAACGCCCTCTTAGAGAGCGTCACTAAGTTCCACTGCGGGGTTATCTACGACTACTCCACCGCTGAGTACGTAAGCTACCGTCCGAGTGACTTCGGTGCGTATCTGGATGCGCTGGAAGCCGAGGTTGCACGAGGCGGTCTTATTGTGTTCCACAACGGTCACAAGTATGACGT                                             <  1:324925/301‑1`  
                                                                                                                                                                                                                                                                       `CAACAGACTGGTCCAACGACACTGACTTCTGACAGGATTCTTGACAGTTGTTTCATATGAAGAGATTGTTAAGTCACGATAATCAATAGGAGAAATCAATATGATCGTTTCTGACATCGAAGCTAACGCCCTCTTAGAGAGCGTCACTAAGTTCCACTGCGGGGTTATCTACGACTACTCCACCGCTGAGTACGTAAGCTACCGTCCGAGTAACTTCGGTGCGTATCTGGATGCGCTGGAAGCCGAGGTTGCACGAGGCGGTCTTATTGTGTTCCACAACGGTCACAAGACTGACGTTCCT                                         >  1:44185/1‑301`  
                                                                                                                                                                                                                                                                        `AACAGACTGGTCCAACGACACTGACTTCTGACAGGATTCTTGACAGTTGTTTCATATGAAGAGATTGTTAAGTCACGATAATCAATAGGAGAAATCAATATGATCGTTTCTGACATCGAAGCTAACGCCCTCTTAGAGAGCGTCACTAAGTTCCACTGCGGGGTTATCTACGACTACTCCACCGCTGAGTACGTAAGCTACCGTCCGAGTGACTTCGGTGCGTATCTGGATGCGCTGGAAGCCGAGGTTGCACGAGGCGGTATTATTGTGTTCCACAACGGTCACAAGTATGACGTTCCTG                                        <  1:279370/301‑1`  
                                                                                                                                                                                                                                                                                     `ttatcagagactgTCTGACAGGCTTCTTGACAGTTGTTTCATATGAAGAGATTGTTAAGTCACGATAATCAATAGGAGAAATCAATATGATCGTTTCTGACATCGAAGCTAACGCCCTCTTAGAGAGCGTCACTAAGTTCCACTGCGGGGTTATCTACGACTACTCCACCGCTGAGTACGTAAGCTACCGTCCGAGTGACTTCGGTGCGTATCTGGATGCGCTGGAAGCCGAGGTTGCACGAGGCGGTCTTATTGTGTTCCACAACGGTCACAAGTATGACGTTCCTGCATTGACCAAACT                           <  1:109545/288‑1`  
                                                                                                                                                                                                                                                                                         `ACACTGACTTCTGACAGTATTCTTGACAGTTGTTTCATATGAAGAGATTGTTAAGTCCCGATAATCAATAGGAGAAATCAATATGATCGTTTCTGACATCGAAGCTAACGCCCTCTTAGAGAGCGTCACTAAGTTCCACTGCGGGGTTATCTACGACTACTCCACCGCTGAGTACGTAAGCTACCGTCCGAGTGACTTCGGTGCGTATCTGGATGCGCTGGAAGCCGAGGTGGCACGAGGCGGTCTTATTGTGTTCCTCAACGGTCCCAAGTATGACGTTCCTGCATTGACCAACCTGGCA                       >  1:55242/1‑301`  
                                                                                                                                                                                                                                                                                              `GACTTCTGACAGGATTCTTGACAGTTGTTTCATATTAAGAGATTGTTAAGTCACGATAATCAATAGGAGAAATCAATATGATCGTTTCTGACATCGAAGCTAACGCCCTCTTAGATAGCGTCACTAAGTTCCACTGCGGGGTTATCTACGACTACTCCACCGCTGAGTACGTAAGCTACCGTCCGAGTGACTTCGGTGCGTATCTGGATGCGCTGGAAGCCGAGGTTGCACGAGGCGGTCTTATTGTGTTCCACACCGGTCACAAGTATGACGTTCCTGCATTTACCAAACTTGCAAAGTT                  >  1:319600/1‑301`  
                                                                                                                                                                                                                                                                                                    `TGACAGGATTCTTGACAGTTGTTTCATATGAAGAGATTGTTAAGTCACGATAATCAATAGGAGAAATCAATATTATCGTTTCTGACATCGAAGCTAACGCCCTCTTAGAGAGCGTCACTAAGTTCCACTGCGGGGTTATCTACGACTACTCCACCGCTGAGTACGTAAGCTACCGTCCGAGTGACTTCGGTGCGTATCTGGATGCGCTGGAAGCCGAGGTTGCACGAGGCGGTCTTATTGTGTTCCACAACGGTCACAAGTATGACGTTCCTGCATTGACCAAACTGGCAAAGTTGCAATT            <  1:254535/301‑1`  
                                                                                                                                                                                                                                                                                                     `GACAGGATTCTTGACAGTTGTTTCATATGAAGAGATTGTTAAGTCACGATAATCAATAGGAGAAATCAATATGATCGTTTCTGACATCGAAGCTAACGCCCTCTTAGAGAGCGTCACTAAGTTCCACTGCGGGGTTATCTACGACTACTCCACCGCTGAGTACGTAAGCTACCGTCCGAGTGACTTCGGTGCGTATCTGGATGCGCTGGAAGCCGAGGTTGCACGAGGCGGTCTTATTGTGTTCCACAACGGTCACAAGTATGACGTTCCTGCATTGACCAAACTGGCAAAGTTGCAATTG           <  1:335170/301‑1`  
                                                                                                                                                                                                                                                                                                        `AGGATTCTTGACAGTTGTTTCATATGAAGAGATTGTTAAGTCACGATAATCAATAGGAGAAATCAATATGATCGTTTCTGACATCGAAGCTAACGCCCTCTTAGAGAGCGTCACTAAGTTCCACTGCGGGGTTATCTACGACTACTCCACCGCTGAGTACGTAAGCTACCGTCCGAGTGACTTCGGTGCGTATCTGGATGCGCTGGAAGCCGAGGTTGCACGAGGCGGTCTTATTGTGTTCCACAACGGTCACAAGTATGACGTTCCTGCATTGACCAAACTGGCAAAGTTGCAATTGAAC        <  1:298943/301‑1`  
                                                                                                                                                                                                                                                                                                        `AGGATTCTTGACAGTTGTTTCATATGAAGAGATTGTTAAGTCACGATAATCAAGAGGAGAAATCAATATGATCGTTTCTGACATCGAAGCTAACGCCCTCTTAGAGAGCGTCACTAAGTTCCACTGCGGGGTTATCTACGACTACTCCACCGATGAGTACGTAAGCTACCGTCCGAGTGACTTCGGTGCGTATCTGGATGCGCTGGAAGCCGAGGTTGCACGAGGCGGTCTTATTGTGTTCCACAACGGTCACAAGTATGACGTTCCTGCATTGACCAAACTGGCAAAGTTGCAATTGAAC        <  1:62582/301‑1`  

                                                                                                                                                                                                                                                                                                                                                                                                                                                                                                                                                                                                                             
`AGACAAAGGTAAAGCACATGAGGAAGGTCGCCCCGTTTCTATTACTGACCTACGTGGTTCTGGCGCACTACGCCAACTATCTGATACTATTATTGCCCTTGAGCGTAATCAGCAAGGCGATATGCCTAACCTTGTCCTCGTTCGTATTCTCAAGTGCCGCTTTACTGGTGATACTGGTATCGCTGGCTACATGGAATACAACAAGGAAACCGGATGGCTTGAACCATCAAGTTACTCAGGGGAAGAAGAGTCACACTCAGAGTCAACAGACTGGTCCAACGACACTGACTTCTGACAGGATTCTTGA‑‑‑‑‑‑‑‑‑‑‑‑‑‑‑‑‑‑‑‑‑‑‑‑‑‑‑‑‑‑‑‑‑‑‑‑‑‑‑‑‑‑‑‑‑‑‑‑‑‑‑‑‑‑‑‑‑‑‑‑‑‑‑‑‑‑‑‑‑‑‑‑‑‑‑‑‑‑‑‑‑‑‑‑‑‑‑‑‑‑‑‑‑‑‑‑‑‑‑‑‑‑‑‑‑‑‑‑‑‑‑‑‑‑‑‑‑‑‑‑‑‑‑‑‑‑‑‑‑‑‑‑‑‑‑‑‑‑‑‑‑‑‑‑‑‑‑‑‑‑‑‑‑‑‑‑‑‑‑‑‑‑‑‑‑‑‑‑‑‑‑‑‑‑‑‑‑‑‑‑‑‑‑‑‑‑‑‑‑‑‑‑‑‑‑‑‑‑‑‑‑‑‑‑‑‑‑‑‑‑‑‑‑‑‑‑‑‑‑‑‑‑‑‑‑‑‑‑‑‑‑‑‑‑‑‑‑‑‑‑‑‑‑‑‑‑‑‑‑‑‑‑‑‑‑‑‑‑‑‑‑‑‑‑‑‑‑‑‑‑‑‑‑‑‑‑‑‑‑‑‑‑‑‑‑‑‑‑‑‑‑‑‑‑‑‑  >  NC_001604.1/12971‑13277`  
`‑‑‑‑‑‑‑‑‑‑‑‑‑‑‑‑‑‑‑‑‑‑‑‑‑‑‑‑‑‑‑‑‑‑‑‑‑‑‑‑‑‑‑‑‑‑‑‑‑‑‑‑‑‑‑‑‑‑‑‑‑‑‑‑‑‑‑‑‑‑‑‑‑‑‑‑‑‑‑‑‑‑‑‑‑‑‑‑‑‑‑‑‑‑‑‑‑‑‑‑‑‑‑‑‑‑‑‑‑‑‑‑‑‑‑‑‑‑‑‑‑‑‑‑‑‑‑‑‑‑‑‑‑‑‑‑‑‑‑‑‑‑‑‑‑‑‑‑‑‑‑‑‑‑‑‑‑‑‑‑‑‑‑‑‑‑‑‑‑‑‑‑‑‑‑‑‑‑‑‑‑‑‑‑‑‑‑‑‑‑‑‑‑‑‑‑‑‑‑‑‑‑‑‑‑‑‑‑‑‑‑‑‑‑‑‑‑‑‑‑‑‑‑‑‑‑‑‑‑‑‑‑‑‑‑‑‑‑‑‑‑‑‑‑‑‑‑‑‑‑‑‑‑‑‑‑‑‑‑‑‑‑‑‑‑‑‑‑‑‑‑‑‑‑‑‑‑‑‑‑‑‑‑‑‑‑‑‑‑‑‑‑‑‑‑‑‑‑‑‑ttcttgaCAGTTGTTTCATATGAAGAGATTGTTAAGTCACGATAATCAATAGGAGAAATCAATATGATCGTTTCTGACATCGAAGCTAACGCCCTCTTAGAGAGCGTCACTAAGTTCCACTGCGGGGTTATCTACGACTACTCCACCGCTGAGTACGTAAGCTACCGTCCGAGTGACTTCGGTGCGTATCTGGATGCGCTGGAAGCCGAGGTTGCACGAGGCGGTCTTATTGTGTTCCACAACGGTCACAAGTATGACGTTCCTGCATTGACCAAACTGGCAAAGTTGCAATTGAACCGAGAG  >  NC_001604.1/14297‑14592`

  

Alignment Legend

Aligned base mismatch/match (shaded by quality score): `ATCG/ATCG` `< 3 ≤` `ATCG/ATCG` `< 11 ≤` `ATCG/ATCG` `< 23 ≤` `ATCG/ATCG` `< 34 ≤` `ATCG/ATCG` `< 38 ≤` `ATCG/ATCG`

Unaligned base: `atcg`    Masked matching base: `atcg`    Alignment gap: `‑`    Deleted base: `‑`

Reads not counted as support for junction

read\_name Not counted due to insufficient overlap past the breakpoint.

read\_name Not counted due to not crossing MOB target site duplication.
